# The motivation to be sedentary predicts weight change when sedentary behaviors are reduced

**DOI:** 10.1186/1479-5868-8-13

**Published:** 2011-02-22

**Authors:** Leonard H Epstein, James N Roemmich, Meghan D Cavanaugh, Rocco A Paluch

**Affiliations:** 1 Department of Pediatrics, School of Medicine and Biomedical Sciences, University at Buffalo, Farber Hall, Room G56, 3435 Main Street, Building #26, Buffalo, New York 14214-3000, USA

## Abstract

**Background:**

Obesity is correlated with a sedentary lifestyle, and the motivation to be active or sedentary is correlated with obesity. The present study tests the hypothesis that the motivation to be active or sedentary is correlated with weight change when children reduce their sedentary behavior.

**Methods:**

The motivation to be active or sedentary, changes in weight, and accelerometer assessed physical activity were collected for 55 families with overweight/obese children who participated in a nine-week field study to examine behavior and weight change as a function of reducing sedentary behavior. Children were studied in three 3-week phases, baseline, reduce targeted sedentary behaviors by 25% and reduce targeted sedentary behaviors by 50%. The targeted sedentary behaviors included television, video game playing, video watching, and computer use.

**Results:**

The reinforcing value of sedentary behavior but not physical activity, was correlated with weight change, as losing weight was associated with lower reinforcing value of sedentary behaviors. Reducing sedentary behavior was not associated with a significant change in objectively measured physical activity, suggesting the main way in which reducing sedentary behavior influenced weight change is by complementary changes in energy intake. Estimated energy intake supported the hypothesis that reducing sedentary behaviors influences weight by reducing energy intake.

**Conclusions:**

These data show that the motivation to be sedentary limits the effects of reducing sedentary behavior on weight change in obese children.

**Trial registration:**

ClinicalTrials.gov: NCT00962247

## Introduction

The choice to be active or sedentary depends in part on individual differences in the motivation to be active or sedentary, as well as constraints on access to sedentary or active alternatives [1,2]. The motivation to be active or sedentary can be operationalized by providing children with the choice to be active or sedentary, varying the behavioral costs to obtain access to the alternatives, and quantifying the amount of work or effort the children will do to gain access to the alternatives. This provides an index of the relative reinforcing value of being active (RRV_ACT_) or sedentary (RRV_SED_). The relative reinforcing value of physical activity has been associated with physical activity levels, with children who find physical activity more reinforcing also being the most active [3-5]. In addition, there are strong individual differences in the reinforcing value of physical activity, as obese children find physical activity less reinforcing than leaner children [6].

Reported time spent watching television, as one component of being sedentary, is cross-sectionally correlated with obesity in children and adults [7-10], as well as being a risk factor for the development of obesity in children [11,12]. Given the role of a sedentary lifestyle in weight gain and the development of obesity, research suggests that reducing sedentary behavior may be a valuable tool in prevention [13,14] and treatment of pediatric obesity [15,16]. There are two potential ways in which reducing sedentary behaviors can be associated with weight changes. As sedentary behaviors are reduced, complementary reductions in energy intake may occur, or as sedentary behaviors are reduced, children may substitute physical activity for sedentary behaviors.

Despite the importance of the motivation to be sedentary or active as a predictor of a child's lifestyle choices, there has been no research on how the motivation to be active or sedentary is associated with weight change when sedentary behaviors are reduced. The purpose of this study is to report on how individual differences in the RRV_SED _or RRV_ACT _are correlated with weight loss during an intervention when sedentary behaviors are reduced.

## Methods

### Participants

Participants were 56 overweight/obese, 8-12 year old American children, recruited from flyers, a direct mailing, and a pre-existing database. All of the children were considered to be overweight or at risk for overweight, defined as a Body Mass Index (BMI) percentile adjusted for age and sex at or above the 85^th ^percentile [17]. Criteria for participation included the following; at least one parent agreed to help their child reduce targeted sedentary behaviors, and measure usual physical activity and dietary intake; the participating child must have engaged in at least 18 hours of targeted sedentary behaviors per week; could not participate in swimming and/or weight training for greater than 5 total combined hours per week; no activity restrictions or physical limitations that could interfere with changes in physical activity, such as developmental disability or injury; no psychopathology or developmental disabilities that would limit participation. All procedures and measures were approved by the University at Buffalo Children and Youth Institutional Review Board.

### Design and Procedure

After completing the phone screen families were scheduled for an orientation. During the orientation parents and children completed consent and assent forms, child height and weight were measured, and families were oriented on the TV allowance device, the physical activity monitor, and activity diaries. Interested families were fitted with an accelerometer which was worn on two weekdays and one weekend day.

Families were scheduled for two laboratory sessions. The child's RRV_SED _and RRV_ACT_, were measured during the first session, and the accelerometers were calibrated during the second session using a progressive treadmill test. After laboratory testing, families were scheduled for 5 home visits throughout the nine week intervention, and children were scheduled to wear the accelerometer on three randomly selected days, two weekdays and one weekend day. Children were also instructed to self-monitor time on each sedentary and active behavior to ensure adherence with the experimental manipulation in a seven day diary during the last week of each phase. Activity devices were downloaded at home visits 3-5 and self report diaries were checked for accuracy. Weight was measured at each home visit and height was measured at the last home visit. Reminder phone calls were made to ensure the child wore the activity device on their scheduled day. Parent and child manuals were provided to each family explaining the study goals as well as to provide techniques for praise and reducing sedentary behaviors.

During the first of five home visits, TV Allowance™ devices were connected to each TV and computer in the home, families were trained on using the devices and asked to maintain their usual pattern of sedentary behaviors, physical activity and dietary intake through the baseline phase; activity devices were fitted to the child, the child was trained on recording in the weekly diary; and weight was measured. During the second home visit each device was checked and TV and computer hours were recorded. At the third home visit, eligibility was determined, amount of screen time was calculated from the allowance devices, and the devices were programmed to decrease TV and computer use by 25% for the next three weeks. During the fourth home visit, devices were programmed to decrease TV and computer use by 50% for the next three weeks. Devices were removed at home visit 5.

Two positive reinforcement techniques were used to facilitate adherence to the experimental protocol, praise and monetary reinforcement. Parents were instructed to praise their children when they observed behavior changes in the appropriate direction, and to be very specific in stating what the praise is for and to be consistent in using praise. Families earned up to $325.00 for participation in the 9 week-study. Children earned up to $15/week during the 25% and 50% reduction phases for making the reductions in targeted sedentary behavior ($90), with the amount proportional to the degree of change, with $10 for reaching the decrease goals and an additional 1$ for every hour under their goal up to $5. During the baseline phase families could earn up to $25/week ($75) for completing measurements, and up to $10 per week ($60) for completing measurements during the 25% and 50% reduction phases. Families also earned $100 for completing the study. Families could distribute the family money as they chose.

### Measurement

#### Demographic variables and medical history

Family size, family income, parent educational level and racial/ethnic background were obtained using a standardized questionnaire. Current medical problems, including psychiatric diagnoses and eating disorders were assessed at baseline by parent interview.

#### Weight, height, BMI

Child weight was assessed by use of a Tanita BWB-800P digital scale. Height was assessed using a Digi-Kit digital stadiometer. On the basis of the height and weight data Body Mass Index (BMI) is calculated according to the following formula: (BMI = kg/m^2^). Children were considered overweight if they were at or above the 85^th ^BMI percentile for their age and sex [17].

#### Liking of activities, food, and videos/computer games

Liking of the activities, videos or computer games was measured on 7 point Likert-type scales anchored by 1 (Do not like) to 7 (Like very much) [18].

#### The relative reinforcing value of sedentary behavior (RRV_SED_) and physical activity (RRV_ACT_)

RRV_SED _or RRV_ACT _is assessed by evaluating how hard a participant will work to obtain access to physical versus sedentary activities [1]. The child first sampled each of the four physical activities and four sedentary behaviors for at least two minutes and then rated them on a scale from 1-7. Children were asked to rank the activities, and the highest rated physical activity and sedentary behavior were chosen for the task. The physical activity alternatives included a balance board, a stationary youth mountain bike, a stepper, and a skipping game, while the sedentary alternative included magazines, puzzles, movies, and Playstation™ 2 video games. The child was instructed how to use the computer-generated task to earn points toward their favorite physical or sedentary activity. The computer displayed three squares where shapes rotated and changed color within each square every time a mouse button was pressed. When all of the shapes matched, the participant earned one point. The child worked on one of two computer monitors, one monitor had the physical activity alternative the other had the sedentary alternative. The reinforcement schedules for both components were initially set at FR4 (fixed ratio 4, which means the participant will earn one point after 4 responses). The schedule increased on a progressive ratio schedule that doubled after 5-points were earned on each schedule. (FR4, FR8, FR16, FR32, FR64, FR128, FR256, FR512, FR 1024 and FR2048). For every five points earned, the participant would receive 2-minutes of time to engage in the activity for which they were playing. The child was able to end the session at any time, they were instructed to tell the experimenter they were all finished when they did not want to earn points any longer. The computer recorded the participants' points earned throughout the session. After instructions were given, the experimenter left the room. RRV_SED _and RRV_ACT _were quantified by the OMAX_SED _and the OMAX_ACT_, which is the maximal amount of responding at the highest reinforcement schedule completed. An intercom and a video camera were in the room so that the experimenter could hear and see into the experimental room from an adjoining room.

#### Measurement of television, video and computer game playing at home

Television, VCR/DVD, video game playing, and computer use was measured using the TV Allowance™. The device has a memory which recorded the amount of time that the targeted child and each family member used since the unit was installed. The device has been used in ongoing research in our laboratory, and was used as an important component of a previous study that successfully reduced television watching to prevent the development of obesity in youth [13,19]. At baseline, unlimited TV and computer hours were set on each device, so that study staff could access the total number of hours for television and computer use for each family member. During the 25 and 50% decrease reduction phase the TV Allowance™ was programmed for the sedentary budget for that phase based on baseline amounts. In addition to the TV Allowance™, self-monitoring of sedentary behavior was recorded in a daily habit book which assessed reading, homework and use of hand-held computer games and targeted sedentary behaviors that cannot be quantified in this objective way. Recording was part of the intervention methodology to facilitate the child meeting their behavioral goals during the reduction phases.

#### Physical activity

The objective measure of physical activity was the Actigraph™ activity monitor, a small, unobtrusive unidirectional accelerometer with extensive validation in youth as a measure of physical activity [20-23]. The activity monitor was set to record minute by minute measures of physical activity. The activity monitor was worn during school and non-school waking hours on three days (two weekdays and one weekend day) during the last week of each three week period. If a child did not wear their activity monitor on the scheduled day, a day similar to the missed day was rescheduled. Weekly diaries were used in combination with the activity monitor to indicate what physically active and targeted sedentary behaviors the child was engaging in during the last week of each phase of the experiment. The activity monitors were downloaded to a computer at each home visit and weekly diaries were reviewed during the weekly home visit with the family.

To determine levels of physical activity based on energy expenditure (METS or metabolic equivalents) we individually calibrated each accelerometer based on a progressive treadmill test. The VO_2 _(mL/kg/min) and accelerometer counts/minute were sampled each minute, and the accelerometer values were regressed against VO_2 _values to estimate energy expenditure for different intensities of physical activity. Based on the regression line, rates of accelerometer counts were determined for each participant that corresponded to rest (0 counts), 2 METS (7 mL/kg/min), 3 METS (MVPA, 10.5 mL/kg/min) and 6 METS (VPA, 21 mL/kg/min). Because the accelerometer was only worn for waking hours, and did not include time spent sleeping, we estimated energy expenditure for the remainder of non-accelerometer sampled minutes as expending 1 MET per minute, and computed calories per minute using estimated resting metabolic rate (RMR) using previously published equations for children [24]. Daily RMR calories were converted to RMR calories per minute, then multiplied by number of minutes not sampled by the accelerometer. Energy expenditure estimates while the accelerometer was being worn included RMR component of energy expenditure.

Based on the estimated total daily energy expenditure, we estimated energy intake and changes in energy balance by considering total daily energy expenditure in respect to weight change. If weight was stable over the nine weeks, it was assumed that energy intake = energy expenditure. If children lost weight, it was assumed that one pound of weight loss was equivalent to negative energy balance of 3500 kcals, or 55.6 kcal/day. Similarly, a gain of one pound over the nine weeks would be equivalent to positive energy balance of 55.6 kcal/day. Based on the estimated energy expenditure and observed weight change, estimated energy intake and changes in energy balance were calculated.

### Analytic Plan

Repeated measures analysis of variance was used to assess whether changes in targeted sedentary behavior were established to ensure that the targeted behavior had been manipulated by the intervention. Repeated measures analysis of variance was also used to assess changes in body weight, and physical activity. Pearson product moment correlation coefficients were used to assess predictors of weight change, as well as the relationship between changes in targeted sedentary behaviors and physical activity, as well as the relationship between RRV_ACT _and RRV_SED_, RRV_ACT _and measured physical activity, and RRV_SED _and total sedentary behavior. Significant factors were then studied in regression models controlling for age, sex and minority status. Significant relationships between weight change and RRV_SED _were explored by median splits dividing children into those who decreased or maintained weight (N = 20) versus increased (N = 41) their weight over the nine weeks of observation.

## Results

The average child was 10.7 ± 1.2 years of age, with a height of 57.4 ± 3.7 in, weight of 118.8 ± 30.5 lbs, BMI of 25.0 ± 4.2, and zBMI of 1.8 ± 0.4. Twenty seven (48.2%) of the children were male, and 14 (25%) were non-Caucasian or minority (Table 1). Changes in weight, targeted sedentary behaviors and physical activity are shown across the three phases in Table 2. The average child had a reduction in targeted sedentary behavior from baseline to 25% and 50% reduction phases (F(2, 110) = 285.00, p < 0.0001), with significant changes from baseline to 25% (p < 0.001), with a further significant reduction from 25% to 50% (p < 0.001). There was a reduction in targeted sedentary behavior of 67.6%, with the majority of change (53%) occurring during the initial reduction phase. The changes in sedentary behavior included significant reductions in television watching (F(2,110) = 197.00, p < 0.001) and computer use (F(2,110) = 40.07, p < 0.001). Small, but significant increases in body weight were observed (F(2,110) = 4.84, p < 0.001), with significant changes from baseline to 50% (p = 0.003) phases, but no differences between the 25% and 50% phases (p = 0.20). There was little change in physical activity accelerometer counts (F(2,110) = 0.49, p = 0.61) over phases. In addition, there were no significant changes in average METS (F(2,94) = 0.60, p = 0.55), or in the percentage of time below 2 METS (F(2,94) = 1.61, p = 0.21), above 2 METS (F(2,94) = 1.61, p = 0.21). Amount of time above 3 METS showed a significant decrease over phases (F(2,94) = 4.78, p = 0.01), with decreases from baseline to the 25% (p = 0.03) and 50% (p = 0.02) reduction phases. Estimated energy intake did not significantly change over phases (F(2,110) = 0.49, p = 0.61).

**Table 1:** Characteristics of the sample (N = 56)

Variable	%
Child sex (M/F)	48.2 (27/56)
% Minority	25.0 (14/56)
	Mean (SD)
Age	10.7 ± 1.2
Height (in)	57.4 ± 3.7
Weight (lb)	118.8 ± 30.5
BMI (kg/m^2^)	25.0 ± 4.2
zBMI	1.8 ± 0.4

**Table 2:** Behavior and weight changes during the baseline, 25 and 50% reduction phases (Mean ± SEM)

	Sedentary Behavior Reductions Phases			
**Variable**	**0**	**25%**	**50%**	**p**

Sedentary behavior (hrs/week)	34.9 ± 1.7	16.4 ± 1.0	11.3 ± 0.7	0 > 25 > 50
Television watching (hrs/week)	29.4 ± 1.7	13.9 ± 0.9	9.5 ± 0.6	0 > 25 > 50
Computer games (hrs/week)	5.5 ± 0.8	2.5 ± 0.4	1.8 ± 0.3	0 > 25 > 50
Weight (lbs.)	118.8 ± 4.1	119.2 ± 4.1	119.6 ± 4.1	0 < 50
Physical activity (counts/min)	576.9 ± 23.8	572.4 ± 25.6	555.7 ± 24.3	NS
Average METs	1.60 ± 0.05	1.58 ± 0.04	1.60 ± 0.05	NS
% time below 2 METS	83.3 ± 2.4	84.6 ± 2.4	84.5 ± 2.4	NS
% time above 2 METS	16.7 ± 2.4	15.4 ± 2.4	15.5 ± 2.4	NS
% time above 3 METS	4.2 ± 0.7	3.5 ± 0.6	3.5 ± 0.6	0 > 25,50
Estimated energy intake	2106.4 ± 65.7	2173.5 ± 65.2	2144.3 ± 74.6	NS

Variables related to weight change included only OMAX_SED _(r = 0.31, p = 0.022). Child age (p = .71), sex (p = .64), minority status (p = .67), income (p = .38), reinforcing value of physical activity (p = .72) or baseline values of weight (p = .45), changes in targeted sedentary behavior (p = .44) or changes in physical activity (p = .15) were not related to weight change. Multiple regression controlling for child age, sex and minority status did not reduce the impact of RRV_SED _on weight change (p = 0.035).

**Figure 1:**
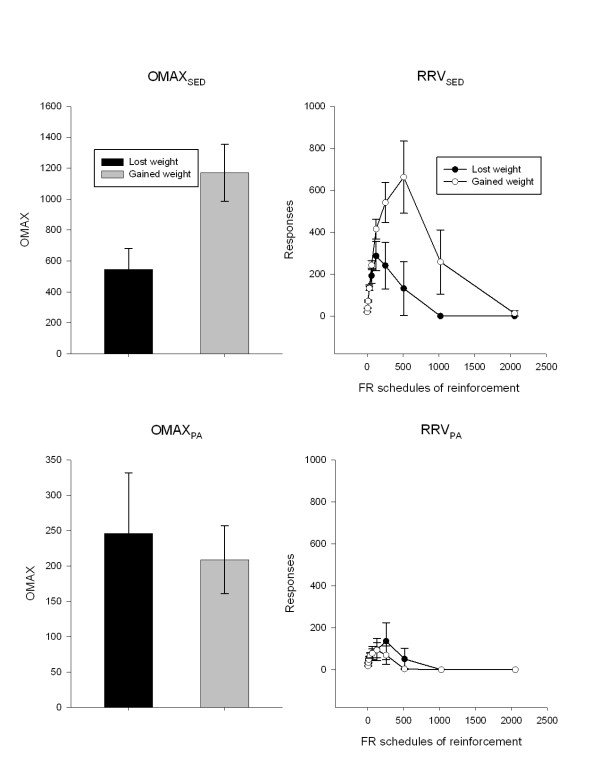
**Differences (mean ± SEM) in the OMAX (left graphs) and reinforcing value (right graphs) of sedentary behaviors (top graphs) or physical activity (bottom graphs) for children who lost or maintained weight versus gained weight during the 9 week study**.

Differences in the motivation to be active or sedentary, and changes in sedentary and active behaviors, estimated energy intake and body weight for children who gained (N = 39) or lost (N = 19) weight are shown in Table 3. There was a significant difference in OMAX_SED _between children who lost or maintained versus those who gained weight during the study (F(1,54 = 4.79, p = 0.03). Figure 1 shows differences in OMAX (left graphs) and the pattern of responding (right graphs), while the top and bottom graphs show motivated responding for sedentary behaviors or physical activity, respectively. As shown in Figure 1, children who maintained or lost weight had lower OMAX_SED _and responding over progressive ratio schedules for access to sedentary behaviors than children who gained weight, who worked much harder for sedentary behaviors. There were no differences in OMAX_ACT _(F(1,54) = 0.11, p = 0.74) as a function of whether children lost or maintained versus gained weight during the study. As shown in Table 3, there were no differences in the alterations in any activity variable for children who gained or lost weight over the nine weeks of the study. Children who lost weight reduced energy intake by an estimated 223 kcal/day calories when sedentary behaviors were reduced, while children who gained weight when sedentary behaviors were reduced increased their estimated energy intake by 172 kcal/day (F(1,54) = 13.13, p = 0.0006).

**Table 3:** Differences in behavior for children who lost or gained weight after reduction of sedentary behaviors (Mean ± SEM)

	Weight change groups		
**Variable**	**Lost (N = 19)**	**Gained (N = 37)**	**p**

OMAX_SED_	540.4 ± 143.5	1189.0 ± 198.4	.03
OMAX_ACT_	258.9 ± 89.4	226.6 ± 52.3	.74
Sedentary behavior (hrs/week)	-26.3 ± 2.0	-22.2 ± 1.6	.13
Television watching (hrs/week)	-22.8 ± 2.1	-18.4 ± 1.6	.12
Computer games (hrs/week)	-3.5 ± 0.8	-3.8 ± 0.8	.83
Weight (lbs.)	-1.2 ± 0.2	1.8 ± 0.2	< .0001
Physical activity (counts/min)	20.6 ± 37.4	-42.5 ± 30.6	.22
Average METs	-0.02 ± 0.03	-.01 ± 0.03	.78
% time below 2 METS	1.45 ± 1.42	1.10 ± 1.09	.85
% time above 2 METS	-1.45 ± 1.42	-1.10 ± 1.09	.85
% time above 3 METS	-0.81 ± 0.6	-0.78 ± 0.4	.96
Estimated energy intake (kcal)	-223.2 ± 105.4	172.0 ± 56.6	.0006

OMAX_ACT _was not significantly related to OMAX_SED _(r = 0.07, p = 0.63). Similarly, OMAX_ACT _was not correlated with activity counts at baseline (r = 0.12, p = 0.38), and OMAX_SED _was not correlated with total sedentary behavior at baseline (r = 0.07, p = 0.63).

## Discussion

The results show that sedentary behavior was successfully manipulated by over 50%, and that children made the majority of the changes during the 25% reduction phase. These changes were associated with no significant increases in physical activity. We have previously observed minimal changes in physical activity when sedentary behaviors were reduced [25]. Weight change was not associated with changes in physical activity when sedentary behaviors are reduced, suggesting that for the average overweight child, reducing sedentary behaviors does not result in greater physical activity and weight loss. This is consistent with cross-sectional research arguing that the effects of sedentary behavior on body weight are not due to changes in activity energy expenditure [26]. In other research we have shown that increasing sedentary behavior results in a reduction in physical activity, suggesting that the relationship between sedentary behavior and physical activity is not symmetric, and the association between these behaviors may only be present in one direction [25].

The variable that was associated with weight change over time was the motivation to be sedentary, which represents the reinforcing value of sedentary behaviors such as television watching, watching videos and playing on the computer. These are activities for which obese children allocate a great deal of their time, and time being sedentary may be independent of time being active [27,28], as the motivation to be sedentary is independent of the motivation to be active. If they were direct substitutes for each other, then they would be significantly negatively correlated, and reducing sedentary behaviors would result in an increase in physical activity, which is generally not observed. In the current study the motivation to be active and the motivation to be sedentary were not significantly related.

It is important that only a subset of sedentary behaviors was targeted, to allow the child to choose how to reallocate time that had been allocated for watching television or playing computer games. Since no increases in physical activity were observed, it is likely that children substituted other sedentary behaviors for the targeted sedentary behaviors. Unfortunately, we did not have children record all sedentary behaviors during the reduction phases, so it is not possible to know what sedentary behaviors they engaged in as substitutes.

The question is how is the reinforcing value of sedentary behaviors related to weight change? Weight change is due to change in energy balance, which must be due to either reductions in energy intake or increases in energy expenditure. The absence of increases in physical activity reduces the likelihood that physical activity is a substitute for sedentary behaviors, such that children spontaneously increase their physical activity when sedentary behaviors are reduced. This suggests that changes in energy intake are the component of energy balance that is promoting the weight change. The estimated energy intake data showed that children who lost weight reduced their estimated daily energy intake by 223 kcal/day, while those who gained weight increased their estimated daily energy intake by 172 kcal/day. Other investigators have argued that while sedentary behaviors are correlated with weight, the relationship is not mediated by changes in measured physical activity, but are likely to be mediated by changes in eating and energy intake [26]. We have previously shown in non-overweight children that energy intake is a reliable complement to shifts in sedentary behavior [19], such that reductions in sedentary behavior paired with eating result in reductions in energy intake. This may be due to the strength of the relationship between eating and engaging in sedentary behaviors when children enter the study. While eating in association with sedentary behaviors is common, and experimental research has shown that increasing television watching increases energy intake [29-31], there is variability in this relationship. If a child never eats in association with television watching, then reducing television watching cannot result in a reduction in energy intake. On the other hand, if a child consumes food often in association with watching television, then reducing television watching may have a large effect on energy intake and body weight.

The motivation to be sedentary is a behavioral phenotype that may lead to a better understanding of factors related to how changing sedentary behavior may relate to changes in energy balance behaviors and weight loss. Given the potential relationship between changes in television watching and energy intake, it is also possible that the motivation to eat is an important factor to predict how reducing sedentary behaviors influences eating. For example, it may be that children who find food more reinforcing would have a harder time reducing food intake when sedentary behaviors are reduced, and they may compensate by increasing intake at other times, or they may resist changes to reduce television watching that is associated with eating, since this would reduce access to powerful reinforcers they want to obtain. This would be an interesting set of studies for future research.

One surprising result was the failure to show that the motivation to be active was related to physical activity, or those high in the motivation to be active were more likely to become more active. We have shown in previous research that the motivation to be active is related to more physical activity [3,4], but these studies included children with a wide variety of motivation to be active as well as a variety of levels of physical activity. In the present study of overweight and obese, sedentary children, the range of motivation to be active and of activity levels was constrained, which can lead to lower relationships if there is little variability in the predictor and/or outcome.

There are limitations to this study in the measurement of activity and diet. While we had objective measurements of physical activity, we did not collect detailed self-reported information on what types of behaviors people engaged in and what types of behaviors people used to substitute for reduced targeted sedentary behaviors. It would have been interesting to know if specific classes of active or sedentary behaviors were changed. For example, recent data suggests that standing rather than sitting may confer health benefits in adults [32], and it would be interesting to know if children stood more or engaged in light physical activity to replace sedentary behaviors and if these changes would be associated with weight loss or improvement in health. Likewise, it would have been interesting to know if other popular sedentary behaviors, such as talking on the phone or texting, replaced television watching or computer game playing. The TV Allowance™ is a useful behavioral engineering approach to reducing television watching, however, a limitation of using the device is that it is possible to overestimate television watching since children may turn on the television and become engaged in an alternative activity and not watch it. This should be minimized since children are reinforced for reducing television watching, but there may be instances in which television watching is overestimated, and the degree of reduction in television watching is underestimated. Physical activity was measured for three days during each phase, and it may have been useful to collect more extended samples of physical activity during each phase [23]. The results point to changes in energy intake, rather than physical activity, as the mechanism for changes in body weight as people reduce their television watching or computer game playing. Estimates of energy intake were consistent with this hypothesis, and accelerometer based activity counts can provide valid information about energy expenditure [20,33]. In addition, self-reports of energy intake are notoriously inaccurate [34], and previous studies using the current methods for reducing sedentary behavior have shown consistent underreporting of energy intake in obese children and adolescents [19]. Despite the challenges in collecting valid dietary intake data, it would be useful to have dietary information that includes dietary intake as well as macronutrient intake.

In summary, the present study replicates previous research that suggests that reducing sedentary behaviors is not associated with an increase in physical activity. The motivation to be sedentary is related to short term weight change when sedentary behaviors are reduced, and this effect may be mediated by changes in energy intake. Thus, one predictor of the effectiveness of programs to reduce sedentary behavior for child weight change may be the motivation to be sedentary.

## Competing interests

Dr. Epstein is a consultant to Kraft foods and NuVal. The other authors do not have any potential conflict of interests.

## Authors' contributions

LHE and JNR designed the study, and LHE obtained the research funding. MDC obtained IRB approval, and supervised study implementation and data collection. LHE and RAP conducted data analysis. LHE wrote the initial draft of the manuscript, and all authors contributed to the interpretation of data and the writing of the manuscript. All authors read and approved the final manuscript.
